# Expression, mutation and copy number analysis of platelet-derived growth factor receptor A (PDGFRA) and its ligand PDGFA in gliomas

**DOI:** 10.1038/sj.bjc.6605225

**Published:** 2009-08-25

**Authors:** O Martinho, A Longatto-Filho, M B K Lambros, A Martins, C Pinheiro, A Silva, F Pardal, J Amorim, A Mackay, F Milanezi, N Tamber, K Fenwick, A Ashworth, J S Reis-Filho, J M Lopes, R M Reis

**Affiliations:** 1 Life and Health Sciences Research Institute (ICVS), School of Health Sciences, University of Minho, 4710 Braga, Portugal; 2 Instituto Adolfo Lutz, 355-01246-902 São Paulo, Brazil; 3 The Breakthrough Breast Cancer Research Centre, Institute of Cancer Research, London SW3 6JB, UK; 4 Department of Pathology, S. Marcos Hospital, 4710 Braga, Portugal; 5 Department of Oncology, S. Marcos Hospital, 4710 Braga, Portugal; 6 IPATIMUP, 4200 Porto, Portugal; 7 Medical Faculties of Porto University, 4200 Porto, Portugal

**Keywords:** PDGFA, PDGFRA, expression, mutations, amplification, gliomas

## Abstract

**Background:**

Malignant gliomas are the most prevalent type of primary brain tumours but the therapeutic armamentarium for these tumours is limited. Platelet-derived growth factor (PDGF) signalling has been shown to be a key regulator of glioma development. Clinical trials evaluating the efficacy of anti-PDGFRA therapies on gliomas are ongoing. In this study, we intended to analyse the expression of PDGFA and its receptor PDGFRA, as well as the underlying genetic (mutations and amplification) mechanisms driving their expression in a large series of human gliomas.

**Methods:**

PDGFA and PDGFRA expression was evaluated by immunohistochemistry in a series of 160 gliomas of distinct World Health Organization (WHO) malignancy grade. *PDGFRA*-activating gene mutations (exons 12, 18 and 23) were assessed in a subset of 86 cases by PCR—single-strand conformational polymorphism (PCR-SSCP), followed by direct sequencing. *PDGFRA* gene amplification analysis was performed in 57 cases by quantitative real-time PCR (QPCR) and further validated in a subset of cases by chromogenic *in situ* hybridisation (CISH) and microarray-based comparative genomic hybridisation (aCGH).

**Results:**

PDGFA and PDGFRA expression was found in 81.2% (130 out of 160) and 29.6% (48 out of 160) of gliomas, respectively. Its expression was significantly correlated with histological type of the tumours; however, no significant association between the expression of the ligand and its receptor was observed. The absence of PDGFA expression was significantly associated with the age of patients and with poor prognosis. Although *PDGFRA* gene-activating mutations were not found, *PDGFRA* gene amplification was observed in 21.1% (12 out of 57) of gliomas. No association was found between the presence of *PDGFRA* gene amplification and expression, excepting for grade II diffuse astrocytomas.

**Conclusion:**

The concurrent expression of PDGFA and PDGFRA in different subtypes of gliomas, reinforce the recognised significance of this signalling pathway in gliomas. *PDGFRA* gene amplification rather than gene mutation may be the underlying genetic mechanism driving PDGFRA overexpression in a portion of gliomas. Taken together, our results could provide in the future a molecular basis for PDGFRA-targeted therapies in gliomas.

Malignant gliomas are highly heterogeneous and invasive tumours and account for approximately 70% of all primary brain tumours (Louis *et al*, 2007). Histologically, gliomas are classified into several entities, with astrocytic tumours being the most prevalent type, followed by oligodendroglial and mixed oligoastrocytic tumours, and less frequently ependymomas (Louis *et al*, 2007). According to the World Health Organisation (WHO), tumours are classified into four grades of malignancy: grade I generally behave in a benign fashion, whereas grade II–IV are biologically malignant, diffusely infiltrating the adjacent brain tissues and ultimately progressing to glioblastoma (WHO grade IV) (Louis *et al*, 2007). Although relatively uncommon, malignant gliomas are associated with disproportionately high morbidity and mortality, with a median survival time of 12 to 15 months for glioblastomas and 24 to 60 months for patients with anaplastic gliomas (Lacroix *et al*, 2001). Despite advances in understanding glioma molecular pathogenesis and treatment improvements, little is known about the cause of this disease and strategies which may result in effective treatment (Louis, 2006; Wen and Kesari, 2008). Therefore, further investigation of the molecular basis of gliomagenesis is essential for the identification of new therapeutic targets for these tumours.

The platelet-derived growth factor receptor A (PDGFRA) is a transmembrane protein with five immunoglobulin-like repeats in the extracellular domain and with a split intracellular tyrosine kinase domain. PDGFRA belongs to class III family of receptor tyrosine kinases (RTKs) that also includes PDGFRB, KIT, the macrophage colony-stimulating-factor receptor and Fl cytokine receptor (Blume-Jensen and Hunter, 2001). Ligand-activated receptors trigger downstream signal transduction pathways, including MAP kinase, PI3-kinase/AKT and JAK/STAT and have pivotal roles in proliferation, differentiation, invasion and survival (Blume-Jensen and Hunter, 2001). PDGFRA and its main ligand PDGFA are key regulators of glial cells proliferation, mainly oligodendrocytes, and have an important role in normal development of the central nervous system (Richardson *et al*, 1988).

Platelet-derived growth factor (PDGF) has also been implicated in cancer, including central nervous system tumours (Shih and Holland, 2006). PDGF and PDGF receptors are commonly coexpressed in gliomas, suggesting that autocrine PDGF receptor stimulation may contribute to their growth (Hermanson *et al*, 1992; Westermark *et al*, 1995). Glioma-like tumours can be induced in mice after overproduction of PDGF in mouse brain (Uhrbom *et al*, 1998). Taken together, these findings provide strong circumstantial evidence to suggest that PDGFR signalling may be a driver of gliomagenesis. Given that PDGFRA is a transmembrane tyrosine kinase receptor and that these receptors have been shown to be amenable to exploitation as therapeutic targets, it seems reasonable to hypothesise that PDGFRA may constitute a potential target for anticancer therapy in gliomas.

The interest in PDGFR as a cancer drug target has increased with the availability of clinically useful small-molecule inhibitors, such as imatinib mesylate (Glivec) and sunitinib (Sutent) (Pietras *et al*, 2003). Imatinib is an orally available RTK inhibitor, which, in addition to PDGFRs, also inhibits KIT, c-Abl, Bcr–Abl and Arg (Capdeville *et al*, 2002). The clinical efficacy of imatinib is well demonstrated in chronic myeloid leukaemia and in gastrointestinal stromal tumours (GISTs), which are driven by activated forms of *BCR–ABL* and mutated *KIT* or *PDGFRA* genes, respectively (Druker *et al*, 2001; Demetri *et al*, 2002). In addition, clinical trials are ongoing using imatinib for the treatment of recurrent glioblastoma patients (Reardon *et al*, 2005; Raymond *et al*, 2008). However, the molecular alterations underlying PDGF overexpression and response to PDGFR antagonists in gliomas remain poorly understood. Thus, the aim of this study was to define the frequency of PDGFRA and PDGFA expression in a large series of gliomas and to determine whether expression of PDGFRA is driven by *PDGFRA* gene mutations and/ or amplification.

## Materials and methods

### Tissue samples

Representative formalin-fixed paraffin-embedded blocks from one hundred and sixty consecutive craniotomies for gliomas were retrieved from pathology archives of the Department of Pathology of Hospital S João, Porto and of Hospital S Marcos, Braga, Portugal. Cases were classified according to the WHO criteria (Louis *et al*, 2007). This series (Table 1) includes 83 astrocytic, 68 oligodendroglial and 9 oligoastrocytic tumours. The mean age of patients at diagnosis was 45.9±17.6 (range, 2–79 years), with a female/male ratio of 0.93. Follow-up data were available in 108 patients (range: 0–210 months, mean: 38.4±42.1 months). The procedures followed in the present study were in accordance with the institutional ethical committees. All the samples enrolled in this study were completely anonymised after retrieval of follow up information.

### PDGFA and PDGFRA immunohistochemistry

Representative 3-*μ*m thick sections were cut from formalin-fixed and paraffin-embedded samples and subjected to immunohistochemical analysis. Immunohistochemistry was carried out using a LabVision Autostainer (LabVision Corporation, Fremont, CA, USA) and the streptavidin—biotin–peroxidase complex technique, with rabbit polyclonal antibodies raised against human PDGFA (clone N-30, dilution 1:80; Santa Cruz Biotechnology, Santa Cruz, USA), and PDGFRA (dilution 1:175; LabVision Corporation) as previously described (Carvalho *et al*, 2005; Reis *et al*, 2005). In brief, deparaffinised and rehydrated sections used to study PDGFA expression were pre-treated by microwaving in 10 mM citrate buffer (pH 6.0) three times for 5 min at 600 W. The sections used for PDGFRA expression were submitted to heat-induced antigen retrieval with 10 mM citrate buffer (pH 6.0) for 20 min in a water bath. After incubation of PDGFA and PDGFRA primary antibody at room temperature for 30 min, the secondary biotinylated goat anti-polyvalent antibody was applied for 10 min followed by incubation with streptavidin–peroxidase complex. The immune reaction was visualised by DAB as a chromogen (Ultravision Detection System Anti-polyvalent, HRP/DAB; LabVision Corporation). Appropriated positive and negative controls were included in each run: for PDGFA and PDGFRA, cutaneous-mucosa transition of the anal region, namely medium calibre vessels with a muscular layer were used as positive controls. For negative controls, primary antibodies were omitted. All sections were counterstained with Gill-2 haematoxylin. As previously described (Reis *et al*, 2005), both the distribution and intense immunoreactivity were semi-quantitatively scored by JML and ALF independently with the observers blinded to the clinical information and results of the other molecular tests as follows: (−) (negative), (+) (⩽5%), (++) (5–50%), and (+++) (>50%). Samples with scores (−) and (+) were considered negative, and those with scores (++) and (+++) were considered positive.

### DNA isolation

Serial 10 *μ*m unstained section of paraffin blocks were cut, and one adjacent haematoxylin and eosin-stained (H&E) section was taken for identification and selection of the tumour tissue. Selected areas containing at least 85% of tumour were marked and macroscopically dissected using a sterile needle (Neolus, 25G–0.5 mm). Tissue was placed into a microfuge tube and DNA isolation was performed using QIAamp DNA Micro Kit (Qiagen, Hilden, Germany) as previously described (Basto *et al*, 2005).

### *PDGFRA* mutations

Pre-screening for mutations in exons 12, 18 and 23 of the *PDGFRA* gene was carried out by PCR-single-strand conformational polymorphism (PCR–SSCP) followed by direct DNA sequencing of samples that showed a mobility shift in the PCR–SSCP analysis, as previously described (Reis *et al*, 2005). Briefly, PCR was carried out in a total volume of 25 *μ*l, consisting of 1–2 *μ*l of DNA solution, 0.5 *μ*M of both sense and anti-sense primers, 200 *μ*M of dNTPs (Fermentas Inc., Glen Burnie, MD, USA), 1.5–2 mM of MgCl2 (Bioron GmbH, Ludwigshafen, Germany), 1 × Taq Buffer Incomplete (Bioron GmbH) and 1U of Taq Superhot DNA Polymerase (Bioron GmbH). The reaction consisted of an initial denaturation at 96 °C for 10 min, followed by 40 cycles with denaturation at 96 °C for 45 s, annealing at 56–60 °C for 45 s and extension at 72 °C for 45 s, followed by a final extension for 10 min at 72 °C, in a Thermocycler (BioRad, Hercules, CA, USA). Primer sequences for exons 12 and 18 were previously reported (Reis *et al*, 2005), and for exon 23 were 5′-GCTCTTCTCTCCCTCCTCCA-3′ (sense) and 5′-TTTCTGAACGGGATCCAGAG-3′ (antisense). PCR products were mixed with an equivalent volume of the denaturing loading buffer (98% formamide, 0.05% xylene cyanol and bromophenol blue). After denaturation at 98 °C for 10 min and quenching on ice, 20 *μ*l of the mixture were loaded onto a gel containing 0.8 × MDE (Cambrex Corporation, East Rutherford, NJ, USA) for exons 12 and 18 and 1 × MDE for exon 23, and 0–3% Glycerol (0% for exon 12 and 3% for exons 18 and 23). The gels were run for 20 h at 4 °C for exons 12 and 18 and 20 °C for exon 23. After the run, the gel was stained with Sybr Gold (Invitrogen Ltd., Paisley, UK) and visualised under ultraviolet light in a UV transilluminator.

Samples showing a mobility shift in the PCR–SSCP analysis different from the normal pattern were directly sequenced (Stab Vida, Investigation and Services in Biological Sciences Lda, Oeiras, Portugal) as previously described (Gomes *et al*, 2007). All positive cases were confirmed twice with a new and independent PCR amplification, followed by direct sequencing.

### Analysis of *PDGFRA* gene copy number status

#### Quantitative real-time PCR

Quantitative real-time PCR (QPCR) was performed with LightCycler (Roche Molecular Biochemicals, Mannheim, Germany), using fluorescent hybridisation probes and fluorescence resonance energy transfer for the detection of PCR amplification product, following the manufacturer's instructions. Briefly, primers and probes were designed to amplify a 124 bp (exon 18 from *PDGFRA* gene), and a 147 bp (*18S* gene) specific PCR product, where 18S was used as reference gene. PCR amplification was performed in a 10 *μ*l reaction volume, under the following conditions: 1 × reaction master mix (Lightcycler FastStart DNA Master Hybridisation Probes kit, Roche Molecular Biochemicals); 0.2 *μ*M Probes (Roche Molecular Biochemicals); 0.5 *μ*M primers; 4 mM MgCl2 (Roche Molecular Biochemicals) and 1 *μ*l (20 ng *μ*l^−1^) of DNA. The reaction was initiated by a denaturation step for 10 min at 95 °C, followed by 45 cycles with the following profile of amplification: incubation for 10 s at 94 °C, specific annealing temperature (57 °C for both genes) for 10 s and extension at 72 °C for an amplicon dependent time (7 s for 18S and 5 s for *PDGFRA*), immediately followed by a cooling step for 2 min at 40 °C. Primers and probes for *18S* gene were previously described (Gomes *et al*, 2007), for *PDGFRA* were as follow: 5′-TCAGCTACAGATGGCTTGATCC-3′ (forward primer), 5′-GCCAAAGTCACAGATCTTCACAAT-3′ (reverse primer), 5′-TGTGTCCACCGTGATCTGGCTGC-FL (donator probe), LC640-CGCAACGTCCTCCTGGCACAAGG-3′ (acceptor probe). The PCR was performed in duplicate for each studied sample. A series of 10 normal DNA from healthy individuals was investigated to determine the confidence interval and the s.d. of the calculated ratios for reference and target gene. Evaluation of data was carried out using the ΔΔ*C*_t_ method: ΔΔ*C*_t_=Δ*C*_t_ Tumour DNA – Δ*C*_t_ Normal blood DNA. Δ*C*_t_ (threshold cycles) is the *C*_t_ of the reference gene minus the *C*_t_ of the target gene. Fold increase of the target gene *PDGFRA* was calculated by 2^(ΔΔCt)^ and values >2 and <5 were defined as aneuploidy and values ⩾5 were considered as gene amplification.

#### Chromogenic *in situ* hybridisation

The presence of *PDGFRA* gene amplification was also assessed by means of chromogenic *in situ* hybridisation (CISH) with an in-house generated probe made up with three contiguous, FISH-mapped and end-sequence verified bacterial artificial chromosomes (BACs) (RP11-626H04, RP11-231C18 and RP11-545H22), which map to the *PDGFRA* locus on 4q12 according to Ensembl V39—June 2006 build of the genome (http://www.ensembl.org/Homo_sapiens/index.html). The in-house probe was generated, biotin-labelled and used in hybridisations as previously described (Lambros *et al*, 2006; Gomes *et al*, 2007). Briefly, heat pre-treatment of deparaffinised sections were incubated for 15 min at 98 °C in CISH pre-treatment buffer (SPOT-light tissue pre-treatment kit, Zymed Laboratories, San Francisco, CA, USA) and digested with pepsin for 6 min at room temperature according to the manufacturer's instructions. CISH experiments were analysed by three of the authors on a multi-headed microscope. Only unequivocal signals were counted. Signals were evaluated at × 400 and × 630 and 60 morphologically unequivocal neoplastic cells were counted for the presence of the gene probe signals. Amplification was defined as >5 signals per nucleus in more than 50% of tumour cells, or when large gene copy clusters were seen (Lambros *et al*, 2006; Gomes *et al*, 2007). CISH hybridisations were evaluated with observers blinded to the clinical information and results of immunohistochemical and QPCR analysis.

#### Microarray-based comparative genomic hybridisation

The aCGH platform used for this study was constructed at the Breakthrough Breast Cancer Research Centre and comprises >16 000 BAC clones tiled across the genome (Arriola *et al*, 2007). This type of BAC array platform has been shown to be as robust as high-density oligonucleotide arrays (Coe *et al*, 2007; Gunnarsson *et al*, 2008) and its actual resolution is approximately 100 kb for >98% of the genome (Arriola *et al*, 2007, 2008; Marchiò *et al*, 2008a). Labelling, hybridisation, washes, image acquisition and data normalisation were carried out as previously described (Arriola *et al*, 2007, 2008; Huang *et al*, 2007; Marchiò *et al*, 2008a, 2008b). Polymorphic BACs identified in an analysis of 50 male/female and female/female hybridisations were filtered out. This left a final dataset of 13711 clones with unambiguous mapping information according to the March 2006 build (hg18) of the human genome (http://www.ensembl.org). Data were smoothed using the adaptive weight smoothing (aws) algorithm (Mackay *et al*, 2009). A categorical analysis was applied to the BACs after classifying them as representing gain, loss, or no-change according to their smoothed Log2 ratio values Threshold values were chosen to correspond to three s.d. of the normal ratios obtained from the filtered clones mapping to chromosomes 1–22, assessed in multiple hybridisations between DNA extracted from a pool of male and female blood donors as previously described (Arriola *et al*, 2008; Reis-Filho *et al*, 2008) (Log2 ratio of ±0.08). Low level gain was defined as a smoothed Log2 ratio of between 0.12 and 0.40, corresponding to approximately 3–5 copies of the locus, whereas gene amplification was defined as having a Log2 ratio >0.40, corresponding to more than 5 copies (Arriola *et al*, 2008). These figures were obtained by comparison with interphase FISH data for markers at different chromosomal locations (Reis-Filho *et al*, 2008). Data processing and analysis were carried out in R 2.0.1 (http://www.r-project.org/) and BioConductor 1.5 (http://www.bioconductor.org/), making extensive use of modified versions of the packages aCGH, marray and aws in particular.

### Statistical analysis

Correlations between categorical variables were performed using the *χ*^2^-test. Cumulative survival probabilities were calculated using the Kaplan–Meier method. Differences between survival rates were tested with the log-rank test. Two-tailed *P*-values <0.05 were considered significant. All statistical analysis was performed using SPSS software for Windows, version 14.0.

## Results

### PDGFA and PDGFRA expression

**Figure 1:**
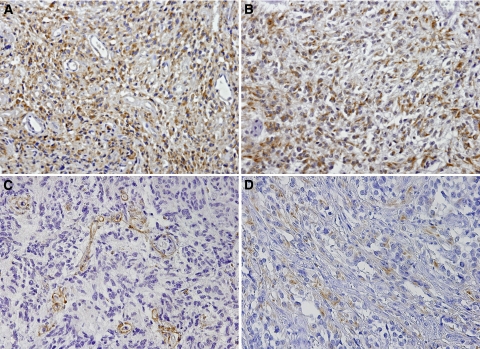
Immunohistochemistry analysis of PDGFA and PDGFRA in gliomas; (**A**) Glioblastoma with (+++) score for PDGFA expression ( × 200); (**B**) Glioblastoma with (+++) score for PDGFRA expression ( × 200). (**C**) Glioblastoma with (−) score for PDGFA expression in tumour cells and positive in endothelial cells ( × 200). (**D**) Glioblastoma with (+) score for PDGFRA expression ( × 200).

PDGFA and PDGFRA expression was found in 81.2% (130 out of 160) and 29.6% (48 out of 160) of gliomas, respectively. Immunohistochemical analysis showed that tumour cells express PDGFA in the cytoplasm (Figure 1A), whereas PDGFRA was preferentially observed in the cytoplasmic compartment and rarely on membranes (Figure 1B). PDGFA expression was also observed in tumour-associated endothelial cells and in basal membrane of blood vessels in approximately 12% (19 out of 160) of the cases (Figure 1C). Representative PDGFRA-negative staining is shown in Figure 1D. Twenty five percent (40 out of 160) of gliomas coexpressed PDGFA and their receptor PDGFRA; however, no statistically significant association between the expression of the ligand and its receptor was observed (*P*>0.05). When the presence or absence of PDGFA/PDGFRA overexpression (+++) was defined according to histological type, a trend for PDGFA and PDGFRA coexpression in glioblastomas was found (*P*=0.069).

PDGFA and PDGFRA expression was significantly correlated with histological type (*P*=0.004 and 0.0001, respectively) (Table 1). When tumours were classified into the three major groups according to their histogenesis (astrocytic, oligodendroglial and mixed), only PDGFRA expression was statistically significantly correlated with astrocytic lineage (*P*<0.001) (Table 1).

In 22 cases, it was possible to analyse PDGFA and PDGFRA expression in both primary and recurrent tumours (Table 2). Overall, the results were concordant; however, we observed loss or gain of PDGFA and PDGFRA in recurrent tumours of some patients (Table 2).

**Figure 2:**
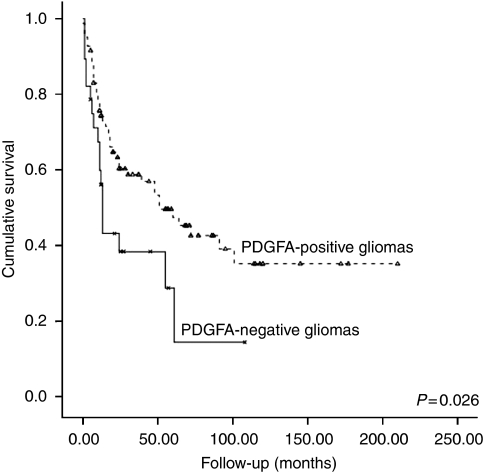
Kaplan–Meier curve illustrating the impact of PDGFA expression on overall survival (months) of glioma patients.

No statistically significant correlations were found between PDGFRA expression and clinical–pathological parameters including age, gender, WHO histological grade and prognosis (Table 1). However, the absence of PDGFA expression was significantly associated (*P*=0.023) with age (>45 years) and with a poor prognosis in glioma patients (*P*=0.026) (Table 1 and Figure 2).

### *PDGFRA* gene mutations

PCR–SSCP analysis for *PDGFRA* gene mutations in exons 12, 18 and 23 produced optimal results in 86 cases, 30 of which were PDGFRA-positive tumours. No activating mutations were found. However, four silent mutations and an intronic insertion were identified in 45.3% (39 out of 86) of glioma patients (Table 3). Five patients showed the simultaneous presence of two different mutations. No association was found between the presence of *PDGFRA* gene mutations and PDGFRA expression (*P*>0.05).

### *PDGFRA* gene amplification

Analysis of *PDGFRA* gene copy number status as defined by QPCR was successfully performed in 57 gliomas. *PDGFRA* copy number changes (ratio >2) were observed in 52.6% (30 out of 57) of glioma patients: 18 displayed ratios <5 and were considered representative of aneuploidy/aneusomy and 12 (21.1%) harboured ratios ⩾5 and were considered amplified (Table 1). No statistically significant associations were found between the *PDGFRA* amplification and PDGFRA expression. However, a borderline association (*P*=0.058) was observed between amplification and overexpression (+++). In addition, a statistically significant association (*P*=0.038) was found between *PDGFRA* amplification and overexpression in grade II diffuse astrocytomas (Table 4). After statistical analysis, no significant correlations were found between the *PDGFRA* gene amplification and clinical–pathological features (*P*>0.05) (Table 1).

In 12 cases, data on *PDGFRA* amplification status in both the primary and recurrent tumours were available (Table 2). In all but one case (tumours 110 and 111) primary and recurrent tumours displayed identical *PDGFRA* copy number status. Interestingly acquisition of *PDGFRA* amplification was observed in the recurrent tumour 111; however, this was not associated with expression of PDGFRA.

**Figure 3:**
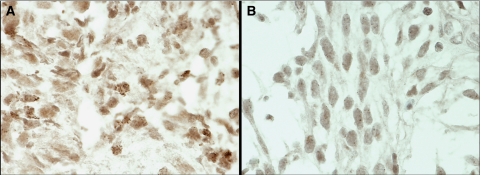
CISH analysis of *PDGFRA* in a glioblastoma (**A**) with *PDGFRA* amplification ( × 600, no HE counterstaining) and other (**B**) without *PDFRA* amplification ( × 600, no HE counterstaining). The colour reproduction of this figure is available on the html full text version of the article.

**Figure 4:**
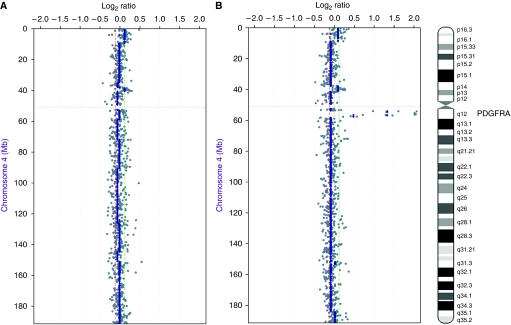
Ideogram and microarray CGH chromosome plots of chromosome 4, in which *PDGFRA* gene is located, for a case without (**A**) and other with (**B**) PDGFRA amplification as defined by QPCR. Log_2_ ratios are plotted on the *x* axis against each clone according to genomic location on the *y* axis. The centromere is represented by a horizontal dotted line. Vertical dashed lines correspond to log_2_ ratios of 0.12 (green) and −0.12 (red). Grey dots: Log_2_ ratios; Blue dots: aws-smoothed Log_2_ ratios. The colour reproduction of this figure is available on the html full text version of the article.

To validate QPCR results we performed CISH in six cases, three with and three without *PDGFRA* amplification as defined by QPCR. CISH analysis of cases defined as harbouring *PDGFRA* gene amplification by QPCR revealed clusters of *PDGFRA* signals in the nuclei of neoplastic cells (Figure 3A), confirming gene amplification. In normal cases, only one-to-two *PDGFRA* gene signals per nucleus were found in neoplastic cells (Figure 3B). To further validate the results of the QPCR analysis, we performed microarray-based comparative genomic hybridisation in two cases, one with and one without *PDGFRA* amplification as defined by QPCR. The case without *PDGFRA* gene amplification by QPCR showed no changes on chromosome 4 (Figure 4A). In the case with amplification, the peak of the amplicon on 4q was restricted to 4q12, encompassing a genomic region of 3.7 Mb (Figure 4B), flanked by the BACs RP11-654K2 (54025,329 kb) and RP11-284L3 (57933,681 kb), including the genes: *LNX1*, *CHIC2*, *GSH2*, *PDGFRA*, *KIT*, *KDR*, *SRD5A2 L*, *TMEM165*, *CLOCK*, *PDCL2*, *NMU*, *EXOC1*, *CEP135*, *AASDH*, *PPAT*, *PAICS*, *SRP72*, *ARL9*, *HOP*, *SPINK2*, *REST*, *C4orf14*, *POLR2B*, and *IGFBP7.* Taken together, the above CISH and aCGH findings provide a robust validation for the results obtained with QPCR.

## Discussion

The PDGF pathway is one of the most consistently altered cellular signalling system in glial tumourigenesis (Shih and Holland, 2006; Fomchenko and Holland, 2007). PDGF and PDGFRs have both been found to be overexpressed in glial tumour cell lines and tumour surgical samples (Nister *et al*, 1988, 1991; Hermanson *et al*, 1992; Black *et al*, 1996; Di Rocco *et al*, 1998; Varela *et al*, 2004). PDGFRA and PDGFA have been shown to be expressed in tumour cells, whereas PDGFB and PDGFRB have been found in glioma-associated endothelial cells (Hermansson *et al*, 1988; Hermanson *et al*, 1992; Plate *et al*, 1992). Furthermore, studies on the PDGFC and D ligands, also demonstrate their expression in gliomas; however, the clinical and biological significance of their expression remain to be determined (Lokker *et al*, 2002). Importantly, the function of PDGF signalling in gliomagenesis has been enlightened by its potential role in cancer stem cell hypothesis of gliomagenesis. It has been suggested that activation of PDGFRA signalling, directly or indirectly through creating a favourable microenvironment niche, can contribute to the transformation of neural stem/progenitor cells into glioma tumours (Jackson *et al*, 2006; Fomchenko and Holland, 2007; Siebzehnrubl *et al*, 2008).

Clinical trials evaluating the efficacy of anti-PDGFRA drugs in patients with glioblastomas are ongoing (Reardon *et al*, 2005; Wen *et al*, 2006; Desjardins *et al*, 2007; Newton, 2007; Raymond *et al*, 2008). Despite the positive response, none of the new targeted therapies has shown significant clinical activity as a single agent in phase II studies (Brandsma and van den Bent, 2007; Raymond *et al*, 2008). Currently, combination of anti-PDGFRA drugs with chemotherapy agents is being evaluated (Reardon *et al*, 2008). However, the molecular alterations underlying glioma patients’ response to PDGFRA antagonists are unknown. One study analysed the prevalence of *PDGFRA* mutations in patients enrolled in a phase I/II study of imatinib mesylate in recurrent malignant gliomas; however, no activating mutations were observed (Wen *et al*, 2006). The *in vitro* studies using PDGFR-targeted drugs (e.g., imatinib) have provided conflicting information about the molecular underpinning of sensitivity to those agents. Although some suggested that sensitivity to targeted agents is associated with overall PDGFR activation; others point to the putative role of PDGFRB or failed to show any association between PDGFR status and response to imatinib (Gross *et al*, 2006; Hägerstrand *et al*, 2006; Servidei *et al*, 2006).

Here, we observed PDGFA expression in 81.2% of gliomas. Overall, PDGFA was highly expressed in all histological types of gliomas. Previous studies on PDGFA mRNA expression reported high levels of PDGFA in gliomas (Maxwell *et al*, 1990; Mapstone, 1991; Hermanson *et al*, 1992; Di Rocco *et al*, 1998). It should be noted, however, that there is a paucity of data on prevalence of PDGFA protein expression in primary glioma specimens. We have previously shown that 100% of gliosarcomas express PDGFA (Reis *et al*, 2005). PDGFRA expression was detected in 29.6% of gliomas, and more frequently expressed in 45–60% of malignant astrocytic tumours. These frequencies are in agreement with previous studies, where approximately 50% for malignant astrocytomas were reported to express this receptor (Nister *et al*, 1988, 1991; Hermanson *et al*, 1992; Black *et al*, 1996; Di Rocco *et al*, 1998; Ribom *et al*, 2002; Varela *et al*, 2004; Liang *et al*, 2008; Takei *et al*, 2008; Thorarinsdottir *et al*, 2008). A previous study showed PDGFRA expression in approximately 50% of gliomas and a correlation with poor prognosis in low-grade gliomas (Varela *et al*, 2004). However, in a report of 40 patients with grade II astrocytomas and oligoastrocytomas, there was an association between high PDGFRA expression and a favourable patient outcome (Ribom *et al*, 2002). Recently, Liang *et al*, (2008) in a paediatric high-grade glioma series failed to find any significant impact of PDGFRA expression on survival. In our series, PDGFRA expression was not correlated with patients’ survival. Interestingly, we found that the absence of PDGFA expression is significantly associated with age and poor prognosis in patients with glioma. Given the retrospective nature of our study, further analysis of the prognostic impact of PDGFA and PDGFRA expression in gliomas is warranted.

Overexpression of RTKs in cancer has been shown to be driven by underlying genetic events in a substantial proportion of cases (Gschwind *et al*, 2004). For instance, KIT overexpression in GISTs is driven by activating *KIT* mutations (Gomes *et al*, 2008), whereas HER2 overexpression in breast cancer is driven by *HER2* gene amplification (Arriola *et al*, 2008). Here, we investigated the prevalence of *PDGFRA*-activating mutations and gene amplification in gliomas. In agreement with previous studies (Hartmann *et al*, 2004; Rand *et al*, 2005; Reis *et al*, 2005; Sihto *et al*, 2005; Wen *et al*, 2006; McLendon *et al*, 2008; Parsons *et al*, 2008), no *PDGFRA*-activating mutations were found. However, four silent mutations and an intronic insertion were identified. Apart from two silent mutations in PDGFRA exon 12, the other mutations have been previously described and considered to be genetic polymorphisms (http://www.ncbi.nlm.nih.gov/projects/SNP/; Carvalho *et al*, 2005; Reis *et al*, 2005; Wen *et al*, 2006). The impact of these genetic polymorphisms in PDGFRA function remains to be elucidated.

*PDGFRA* gene amplification analysis revealed *PDGFRA* amplification in 21.1% (12 out of 57) of gliomas, a frequency similar to that described in previous studies (Fleming *et al*, 1992; Kumabe *et al*, 1992; Smith *et al*, 2000; Alonso *et al*, 2005; Arjona *et al*, 2005; Puputti *et al*, 2006; Beroukhim *et al*, 2007; McLendon *et al*, 2008). We have further shown by aCGH that the amplicon encompasses a region of 3.6 Mb, which, in addition to *PDGFRA*, also includes *KIT* and *KDR* oncogenes. Co-amplification of these three oncogenes has already been detected with other methodologies in gliomas (Joensuu *et al*, 2005; Puputti *et al*, 2006; Holtkamp *et al*, 2007). A statistically significant association between *PDGFRA* amplification and overexpression was found only in diffuse astrocytomas (grade II). Given that PDGFRA overexpression appears to be an early event in gliomagenesis (Hermanson *et al*, 1996), our results provide support to the contention that gene amplification may be one of the underlying mechanisms at this stage. In a way akin to other oncogenes, such as EGFR, overexpression of PDGFRA was more pervasive than gene amplification. It should be noted, however, that there were ∼42% of cases with *PDGFRA* amplification that lacked PDGFRA protein expression, suggesting that in some cases the target of 4q12 amplification may be a gene other than *PDGFRA.*

In conclusion, here we show that PDGFA is expressed in different types of gliomas and its absence is associated with a poor prognosis. PDGFRA is significantly highly expressed in malignant astrocytic tumours. Based on the concurrent expression of PDGFA and PDGFRA in glioblastomas, it could be hypothesised that autocrine/paracrine loops may be present in these tumours, corroborating the importance of this signalling pathway in gliomas (Nister *et al*, 1991; Guha *et al*, 1995; Fomchenko and Holland, 2007). *PDGFRA* gene amplification may be the underlying genetic mechanism driving PDGFRA overexpression in gliomas. However, ∼42% of cases with amplification of *PDGFRA* did not display PDGFRA protein expression, suggesting that a gene other than *PDGFRA* may be the driver of this amplicon. Further studies are needed to correlate these molecular alterations and response to anti-PDGFRA drugs and to investigate the alternative drivers of the 4q12 amplicon. However, our results provide a step forward in the identification of a molecular basis for tailoring the therapies for specific subgroups of glioma patients.

## Figures and Tables

**Table 1:** PDGFA /PDGFRA expression and *PDGFRA* amplification in glioma patients and correlation with clinical–pathological data

	**PDGFA expression (*N*=160)**				**PDGFRA expression (*N*=160)**			***PDGFRA* Amplification (*N*=57)^a^**			
**Parameter**	***N***	**Negative (%)**	**Positive (%)**	***P*-value**	**Negative (%)**	**Positive (%)**	***P*-value**	***N***	**Not amplified (%)**	**Amplified (%)**	***P*-value**
*Age (years)*											
⩾45	83	21 (25.3)	62 (74.7)	0.023^*^	55 (66.3)	28 (33.7)	0.125	36	28 (77.8)	8 (22.2)	0.515
<45	71	8 (11.3)	63 (88.7)		55 (77.5)	16 (22.5)		20	17 (85.0)	3 (15.0)	
											
*Gender*											
Male	82	13 (15.9)	69 (84.1)	0.355	55 (67.1)	27 (32.9)	0.236	33	28 (84.8)	5 (15.2)	0.196
Female	74	16 (21.6)	58 (78.4)		56 (75.7)	18 (24.3)		20	14 (70.0)	6 (30.0)	
											
*Cellular lineage*											
Astrocytic	83	14 (16.9)	69 (83.1)	0.483	45 (54.2)	38 (45.8)	<0.001^*^	32	24 (75.0)	8 (25.0)	0.334
Oligodendroglial	68	13 (19.1)	55 (80.9)		61 (89.7)	7 (10.3)		23	20 (87.0)	3 (13.0)	
Oligoastrocytic	9	3 (33.3)	6 (66.7)		6 (66.7)	3 (33.3)		2	1 (50.0)	1 (50.0)	
											
*Histological type (WHO grade)*											
Pilocytic astrocytoma (I)	9	0 (0)	9 (100)	0.004^*^	8 (88.9)	1 (11.1)	<0.001^*^	1	1 (100)	0 (0)	0.227
Diffuse astrocytoma (II)	33	1 (3.0)	32 (97.0)		18 (54.5)	15 (45.5)		10	5 (50.0)	5 (50.0)	
Anaplastic astrocytoma (III)	5	0 (0)	5 (100)		2 (40.0)	3 (60.0)		2	2 (100)	0 (0)	
Glioblastoma (IV)	36	13 (36.1)	23 (63.9)		17 (47.2)	19 (52.5)		19	16 (84.2)	3 (15.8)	
Oligodendroglioma (II)	32	9 (28.1)	23 (71.9)		28 (87.5)	4 (12.5)		10	9 (90)	1 (10)	
Anaplastic oligodendroglioma (III)	36	4 (11.1)	32 (88.9)		33 (91.7)	3 (8.3)		13	11 (84.6)	2 (15.4)	
Oligoastrocytoma (II)	2	1 (50.0)	1 (50.0)		2 (100)	0 (0)					
Anaplastic oligoastrocytoma (III)	7	2 (28.6)	5 (71.4)		4 (57.1)	3 (42.9)		2	1 (50)	1 (50)	
											
*Malignancy grade (WHO)*											
Low-grade (I and II)	76	11 (14.5)	65 (85.5)	0.187	56 (73.7)	20 (26.3)	0.333	21	15 (71.4)	6 (28.6)	0.232
High-grade (III and IV)	84	19 (22.6)	65 (77.4)		56 (66.7)	28 (33.3)		36	30 (83.3)	6 (16.7)	
											
*Follow-up: mean months±s.d.*											
Gliomas	108	36.1±8.8	99.9±11.9	0.026^*^	87.4±12.1	74.9±15.8	0.664	40	43.9±8.5	53.1±19.4	0.580
Glioblastomas (WHO IV)	29	8.6±1.4	16.7±3.5	0.526	12.1±3.2	11.5±1.9	0.576	16	10.2±1.9	12.0±1.0	0.854

a Assessed by QPCR; N=number of cases; (^*^) Statistically significant values (*P*<0.05).

**Table 2:** PDGFA /PDGFRA expression and *PDGFRA* amplification in glioma patients with recurrences

		**PDGFA expression**		**PDGFRA expression**		***PDGFRA* amplification^a^**	
**Case**	**Classification (P/R)**	**P**	**R**	**P**	**R**	**P**	**R**
58, 59	FA/FA	+	−	+	−	Amplified	Amplified
48, 93	GBM/GBM	+	+	+	+	Normal	Normal
24, 25	GBM/GBM	+	+	+	+	Normal	Normal
168, 169	O/AO	−	+	+	+	Normal	Normal
200, 201	AO/AO	+	+	−	+	Aneuploid	Aneuploid
132, 131	P/P	+	+	−	−	Normal	Normal
28, 52, 53	FA/AA/GBM	+	−/−	+	−/−	ND	ND
32, 35	GBM/GBM	−	−	−	−	ND	ND
30, 39	GBM/GBM	−	+	−	−	ND	ND
88, 89	O/O	+	+	−	−	Normal	Normal
103, 104	O/O	+	+	−	−	Normal	Normal
188, 209, 190	O/AO/AO	+	+/+	−	−/−	ND	ND
185, 186	O/AO	+	+	−	−	Normal	Normal
98, 100	O/AO	+	+	−	−	Normal	Normal
77, 78, 79	O/AO	+	+	−	−	Normal	Normal
181, 182	O/AO	+	+	−	−	ND	ND
177, 178, 179	O/AO/AO	+	+/+	−	−/−	ND	ND
92, 101	AO/AO	+	+	−	+	ND	ND
172, 173	AO/AO	+	+	+	−	ND	ND
165,166	AO/O	−	−	−	−	ND	ND
110, 111	AO/AO	+	+	−	−	Normal	Amplified
160, 161, 162	AO/AO/AO	+	+/+	−	−/−	ND	ND

a Assessed by QPCR; P=Primary tumour; R=Recurrent tumour; P=Pilocytic astrocytoma (grade I); FA=fibrilar astrocytoma (grade II); AA=anaplastic astrocytoma (grade III); GBM=glioblastoma (grade IV); O=oligodendroglioma (grade II); AO=anaplastic oligodendroglioma (grade III); (−)=Negative expression (score 0 and +); (+)=Positive expression (score ++ and +++); ND=not done.

**Table 3:** Sequence variants of *PDGFRA* gene in glioma patients

**Exon**	**Nucleotide change**	**Amino-acid substitution**	**No** **of cases**	**dbSNP**
Exon 12				
	1686 T>C	I562I	1	Not yet described
	1701 G>A	P567P	6	rs1873778
	1777 C>T	L593 L	1	Not yet described
Exon 18				
	2472 C>T	V824V	21	rs2228230
	2449_50insA	IVS18-50insA	15	rs3830355

dbSNP=single nucleotide polymorphism database (http://www.ncbi.nlm.nih.gov/SNP/).

**Table 4:** Correlation between PDGFRA overexpression and *PDGFRA* amplification in gliomas

			***PDGFRA* amplification^a^**		
**Histological type (WHO grade)**	***N***	**PDGFRA overexpression**	**Not amplified (%)**	**Amplified (%)**	***P*-value**
Pilocytic astrocytoma (I)	1	Negative	1 (100)	0 (0)	NP
		overexpression	0 (0)	0 (0)	
Diffuse astrocytoma (II)	10	Negative	5 (100)	2 (40.0)	0.038*
		overexpression	0 (0)	3 (60.0)	
Anaplastic astrocytoma (III)	2	Negative	2 (100)	0 (0)	NP
		overexpression	0 (0)	0 (0)	
Glioblastoma (IV)	19	Negative	4 (25.0)	1 (33.3)	0.624
		overexpression	12 (75.0)	2 (66.7)	
Oligodendroglioma (II)	10	Negative	8 (88.9)	1 (100)	0.900
		overexpression	1 (11.1)	0 (0)	
Anaplastic oligodendroglioma (III)	13	Negative	11 (100)	1 (50)	0.154
		overexpression	0 (0)	1 (50)	
Anaplastic oligoastrocytoma (III)	2	Negative	1 (100)	0 (0)	0.500
		overexpression	0 (0)	1 (100)	

a Assessed by QPCR; Negative expression=scores 0, + and ++; Overexpression=score +++; (^*^) Statistically significant values (*P*<0.05); NP=not possible.
